# Supplementary material for: Effect of Combining Impact-Aerobic and Strength Exercise, and Dietary Habits on Body Composition in Breast Cancer Survivors Treated with Aromatase Inhibitors
Source: Int J Environ Res Public Health. 2023 Mar 10;20(6):4872. doi: 10.3390/ijerph20064872 (PMC10049091; doi:10.3390/ijerph20064872)
Supplement: Supplementary file 1 [file ijerph-20-04872-s001.zip › ijerph-2241281-supplementary.pdf]

Table S1. Classification of nutritional status of participants according to BMI in the initial and final study.

	Initial (n = 43)		Final (n = 43)	
**Nutritional Status, BMI (kg/m^2)**	**n**	**%**	**n**	**%**
Normal weight (BMI: 18.5-24.9)	16	37.2	17	39.5
Overweight (BMI: 25.0-29.9)	18	41.8	17	39.5
Obesity (BMI >30.0)	9	20.9	9	20.9

Notes: BMI —body mass index (kg/m^2)—, n —number of participants—, % —the percentage of total participants—.

Table S2. Changes in biochemical parameters in the Control Group (CG) and Intervention Group (IG) after one year of the study.

	Control Group (CG)					Intervention group (IG)					CG vs. IG		
	Initial		Final		(a)	Initial		Final		(a)	(b)		
BQ	Mean	SD	Mean	SD	p-value	Mean	SD	Mean	SD	p-value	Mean	SD	P♯ p-value
Glucose (mg/dL)	99.6	17.8	98.2	14.5	0.443	98.1	12.6	92.9	7.4	0.068	-2.54	9.01	0.083
Urea (mg/d)	34	12.0	36.1	8.3	0.672	37.5	10.8	35.8	7.3	0.886	0.46	7.82	0.656
Creatinine (mg/d)	0.8	0.1	0.8	0.1	0.167	0.7	0.1	0.7	0.1	0.205	0.01	0.7	0.071
Urates (mg/d)	4.5	1.0	4.9	1.3	0.446	4.3	0.8	5.0	1.4	0.539	0.13	0.71	0.696
Total Cholesterol (mg/dL)	198.9	29.2	208.0	35.3	0.888	227.6	37.1	222.8	31.0	0.374	-3.37	25.95	0.342
Triglycerides (mg/dL)	88.7	29.5	91.1	26.5	1.000	99.7	62.2	104.7	81.5	0.310	6	21.62	0.493
HDL-c (mg/dL)	63.3	17.0	58.7	15.9	0.276	62.3	13.6	59.9	10.9	0.263	-3.9	8.34	0.728
LDL-c (mg/dL)	132.7	31.1	137.1	28.4	0.108	152.4	26.3	143.6	23.4	0.128	-7.92	25.65	0.087
Albumin(g/dL)	4.3	0.4	4.3	0.2	0.345	5.7	5.2	4.3	0.2	0.666	-1.38	4.99	0.894
Alkaline phosphatase (U/L)	76.8	32.3	85.1	28.2	0.068	72.7	23.6	74.6	18.5	0.317	-0.12	18.3	0.068
Calcium (mmol/L)	9.5	0.4	9.7	0.3	0.317	9.0	0.2	9.2	1.9	0.655	-0.02	0.37	0.220
Phosphorus (mmol/L)	3.8	0.5	3.6	0.4	0.655	3.6	0.9	5.1	6.1	0.068	-0.10	0.28	0.439

Notes: (a) —Within-group comparison of differences in biochemical parameters, between the beginning and end of the study, for each parameter and the p-value—, (b) —the comparison of differences in biochemical parameters at the end of the study between CG and IG and the p-value — *P♯*—.

SD —standard deviation—. *Significant difference ($p \leq 0.05$). The significant *p*-value is shown in bold.

Table S3. Level of adherence to the Mediterranean Diet pattern according to the Mediterranean Diet score, in the initial and final study.

	**Initial (n = *43)***					**Final (*n* = *43)***				
	**Control Group (CG)**		**Intervention Group (IG)**			**Control Group (CG)**		**Intervention Group (IG)**		
**Mediterranean Diet Adherence**	**n**	**%**	**n**	**%**	***p-value***	**n**	**%**	**n**	**%**	***p-value***
Low (0-2 score)	2	4.6	0	0	0.198	0	0	2	4.6	0.532
Moderate (3-6 score)	14	32.5	18	41.8		18	41.8	15	34.8	
High (7-9 score)	6	13.9	3	6.9		4	9.3	4	9.3	

Notes: n— number of participants—, % — the percentage of total participants—, *p-value* — differences between the Control Group and Intervention Group, in the initial and final study.*Significant difference ($p \leq 0.05$). The significant *p* values are shown in bold.

Table S4. Energy and macronutrient intake changes after one year of intervention.

	Control Group (CG)							Intervention Group (IG)							CG *vs*. IG
	Initial		Final		(a)			Initial		Final		(a)			(b)
Nutrients	*Mean*	*SD*	*Mean*	*SD*	*Mean*	*SD*	*p-value*	*Mean*	*SD*	*Mean*	*SD*	*Mean*	*SD*	*p-value*	*P#* *p-value*
Energy intake (Kcal/d)	1807.6	384.7	1737.7	376.5	-69.9	267.5	0.234	1667.6	459.8	1596.0	370.3	-71.6	366.0	0.380	0.986
Water (ml/d)	1993.3	548.5	1989.1	625.7	-4.2	376.6	0.286	1966.2	561.6	1850.2	650.1	-115.9	504.4	0.305	0.159
Carbohydrates (g/d)	157.8	48.3	143.1	29.1	-14.7	38.2	0.086	140.4	43.2	148.4	52.3	8.0	32.5	0.273	0.043*
Polysaccharides (g/d)	76.8	31.7	68.1	20.1	-8.7	30.1	0.188	67.33	24.5	69.9	33.1	2.5	34.0	0.738	0.257
Sugar (g/d)	77.9	25.3	74.3	17.7	-3.5	21.4	0.449	71.2	31.8	69.9	28.9	-1.3	21.8	0.790	0.735
Fiber (g/d)	21.8	8.2	20.2	6.0	-1.6	8.4	0.381	16.3	5.7	19.0	9.1	2.6	8.0	0.148	0.099
Total protein (g/d)	79.6	10.4	82.0	22.4	2.4	21.9	0.249	68.4	13.4	74.5	10.5	6.0	13.7	0.057	0.159
Animal protein (g/d)	56.4	11.7	61.41	24.80	5.1	22.9	0.286	47.7	11.0	52.3	11.2	4.6	11.9	0.092	0.331
Plant-based protein (g/d)	23.8	8.6	20.81	4.97	-3.0	7.6	0.083	20.7	6.5	21.4	7.5	0.7	8.0	0.702	0.135
Total fat (g/d)	90.1	24.6	88.01	33.26	-2.11	28.28	0.730	85.89	41.01	75.2	25.5	-10.7	39.7	0.664	0.734
Saturated fatty acids (g/d)	20.9	6.7	21.1	8.1	0.3	4.9	0.817	20.1	8.4	17.4	6.6	-2.7	8.3	0.152	0.170
Monounsaturated fatty acids (g/d)	48.7	16.6	47.2	22.3	-1.5	19.3	0.727	48.9	27.7	39.7	14.4	-9.3	27.8	1.000	0.734
Polyunsaturated fatty acids (g/d)	14.2	3.7	17.4	19.3	3.2	18.2	0.524	11.1	4.4	11.9	5.0	0.7	3.9	0.396	0.274
Cholesterol (mg/d)	294.4	132.7	314.9	139.5	20.5	129.9	0.467	291.0	100.7	290.6	98.7	-0.5	133.7	1.000	0.605
Alcohol (g/d)	6.0	7.4	5.1	7.0	-0.9	5.9	0.774	7.0	11.9	4.9	8.9	-2.1	7.5	0.581	0.705

Notes: (a) —Within-group comparison of differences in nutrient intake, between the beginning and end of the study, for each nutrient—, (b) —the comparison of differences in nutrient intake at the end of the study between CG and IG and the p-value — *P♯*—. *Significant difference ($p \leq 0.05$). The significant *p*-value is shown in bold.

Table S5. Micronutrient intake after one year of intervention.

	Control Group (CG)							Intervention Group (IG)							
	Initial		Final		(a)			Initial		Final		(a)			(b)
Nutrients	*Mean*	*SD*	*Mean*	*SD*	*Mean*	*SD*	*p-value*	*Mean*	*SD*	*Mean*	*SD*	*Mean*	*SD*	*p-value*	*P♯ p-value*
Sodium (mg/d)	2081.8	901.7	1771.7	579.1	-310.0	1042.9	0.524	1671.2	500.1	1933.7	675.33	262.5	621.4	0.067	0.052
Potassium (mg/d)	3321.9	754.5	3126.2	506.6	-195.7	680.5	0.192	2865.7	751.7	2896.7	721.6	30.9	815.9	0.864	0.328
Total Calcium (mg/d)	1289.2	595.4	1612.8	434.3	323.7	569.8	**0.015***	1542.7	430.4	1613.5	403.6	70.8	550.3	0.562	0.147
Dietary Calcium (mg/d)	743.7	199.5	749.2	201.4	5.5	195.5	0.897	757.0	251.9	730.1	209.5	-26.8	315.5	0.701	0.687
Magnesium (mg/d)	330.3	111.5	300.9	56.1	-29.4	90.9	0.144	273.2	66.9	301.0	88.3	27.9	77.5	0.115	**0.032***
Phosphorus (mg/d)	1196.0	196.7	1203.2	239.2	7. 2	284.1	0.907	1029.5	248.1	1151.3	217.1	121.8	284.4	0.064	0.194
Iron (mg/d)	11.7	3.6	11.1	2.3	-0.5	3.2	0.450	9.5	2.3	11.3	3.7	1.9	3.8	**0.035***	**0.030***
Zinc (mg/d)	8.3	1.8	8.2	2.1	-0.1	2.1	0.842	7.2	2.3	7.7	2.0	0.5	2.9	0.189	0.274
Vit A (μg /d)	626.6	344.6	905.3	761.6	278.7	825.4	0.832	720.4	571.7	584.7	168.4	-135.8	601.3	0.664	0.285
Retinoids (μg /d)	188.7	136.2	392.5	759.7	203.7	755.0	1.000	254.6	389.1	584.7	168.4	-330.1	412.92	1.000	0.512
Carotenes (μg /d)	2627.0	1599.3	3110.0	2252.4	483.0	451.6	0.832	2412.6	1029.3	2556.1	881.5	143.4	1046.2	0.537	0.961
TotalVit D (μg/d)	16.0	12.3	22.0	8.4	5.9	13.1	**0.045***	21.1	9.9	23.0	7.8	1.8	11.2	0.464	0.409

Dietary Vit D (μg/d)	4.1	3.4	3.3	3.2	-0.8	4.5	0.422	3.0	2.5	3.3	3.2	0.4	4.1	0.692	0.389
Vit E (mg /d)	13.2	4.7	12.9	5.7	-0.4	5.0	0.134	12.0	4.9	11.1	3.2	-0.9	4.2	0.383	0.416
Thiamine (mg /d)	1.3	0.3	1.3	0.3	0.0	0.5	0.891	1.2	0.4	1.3	0.4	0.1	0.4	0.134	0.358
Riboflavin (mg/d)	1.5	0.3	1.6	0.4	0.1	0.5	0.432	1.5	0.6	1.6	0.6	0.1	0.6	0.273	0.846
Niacin (mg /d)	19.9	4.4	21.6	6.4	1.7	7.7	0.3	16.6	4.7	19.8	4.7	3.2	5.0	**0.007****	0.452
Pyridoxine (mg/d)	1.9	0.4	1.9	0.5	0.0	0.6	0.911	1.7	0.6	1.8	0.5	0.1	0.5	0.365	0.597
Folic acid (μg/d)	324.6	88.5	350.2	79.4	25.6	93.3	0.212	302.4	97.0	306.8	116.9	4.4	116.8	0.865	0.514
Vit B12 (μg/d)	5.1	3.1	6.6	6.6	1.5	6.8	0.664	5.3	4.2	4.3	1.9	-1.0	4.7	1.000	0.681
Vit C (mg/d)	169.2	73.5	164.9	65.1	-4.4	69.2	0.769	162.5	70.2	164.3	93.5	1.8	61.6	0.794	0.865

Notes: (a) —Within-group comparison of differences in nutrient intake, between the beginning and end of the study, for each nutrient—, (b) —the comparison of differences in nutrient intake at the end of the study between CG and IG and the p-value — *P#*—. *Significant difference (*p* ≤0.05). The significant *p*-value is shown in bold.
